# EfficientNetV2 Based Ensemble Model for Quality Estimation of Diabetic Retinopathy Images from DeepDRiD

**DOI:** 10.3390/diagnostics13040622

**Published:** 2023-02-08

**Authors:** Sudhakar Tummala, Venkata Sainath Gupta Thadikemalla, Seifedine Kadry, Mohamed Sharaf, Hafiz Tayyab Rauf

**Affiliations:** 1 Department of Electronics and Communication Engineering, School of Engineering and Sciences, SRM University-AP, Amaravati 522240, Andhra Pradesh, India; 2 Department of Electronics and Communication Engineering, Velagapudi Ramakrishna Siddhartha Engineering College, Vijayawada 520007, Andhra Pradesh, India; 3 Department of Applied Data Science, Noroff University College, 4612 Kristiansand, Norway; 4 Artificial Intelligence Research Center (AIRC), Ajman University, Ajman 346, United Arab Emirates; 5 Department of Electrical and Computer Engineering, Lebanese American University, Byblos P.O. Box 36, Lebanon; 6 Industrial Engineering Department, College of Engineering, King Saud University, P.O. Box 800, Riyadh 11421, Saudi Arabia; 7 Centre for Smart Systems, AI and Cybersecurity, Staffordshire University, Stoke-on-Trent ST4 2DE, UK

**Keywords:** diabetic retinopathy, quality estimation, DeepDRiD, *EfficientNetV2*, fundus image

## Abstract

Diabetic retinopathy (DR) is one of the major complications caused by diabetes and is usually identified from retinal fundus images. Screening of DR from digital fundus images could be time-consuming and error-prone for ophthalmologists. For efficient DR screening, good quality of the fundus image is essential and thereby reduces diagnostic errors. Hence, in this work, an automated method for quality estimation (QE) of digital fundus images using an ensemble of recent state-of-the-art *EfficientNetV2* deep neural network models is proposed. The ensemble method was cross-validated and tested on one of the largest openly available datasets, the Deep Diabetic Retinopathy Image Dataset (DeepDRiD). We obtained a test accuracy of 75% for the QE, outperforming the existing methods on the DeepDRiD. Hence, the proposed ensemble method may be a potential tool for automated QE of fundus images and could be handy to ophthalmologists.

## 1. Introduction

Diabetic retinopathy (DR) is a common disease caused by diabetes, majorly affecting working individuals and leading to loss of vision. By 2040, it is estimated that 600 million people will suffer from diabetes, and approximately one third of them will have a chance of getting DR [1]. An ophthalmologist usually identifies DR by visual examination of digital fundus images for the presence of one or more retinal lesions such as microaneurysms, soft exudates, hemorrhages, and hard exudates [2]. DR can broadly be classified into non-proliferative DR (NPDR) and proliferative DR (PDR). The preliminary stage of DR is NPDR, where the microaneurysms are visible in the digital fundus image, and the advanced stage of DR is PDR which can lead to severe vision loss. The NPDR is further subdivided into three types: mild, moderate, and severe NPDR. The international clinical DR severity scale contains five grades to classify fundus images—grade 0 is no apparent retinopathy, grade five is PDR, and the types mentioned above of NPDR are classified as grade one, two, and three, respectively.

The manual evaluation of fundus images may create a severe burden on ophthalmologists. Moreover, accurate grading of DR requires trained healthcare professionals and manual grading could be prone to errors while handling large amounts of data. Hence, automated methods for DR screening are warranted to reduce diagnostic oversights by ophthalmologists and healthcare practitioners. Furthermore, poor-quality digital fundus images due to uneven illumination, blurring, and other artifacts can lead to false positives. Hence, it is vital to first estimate the quality of acquired funds images before proceeding with DR grading [3]. Therefore, fully automated methods for accurate quality estimation (QE) of digital fundus images are in demand since the ratio of doctors to patients is deteriorating. Overall, there is a need for objective evaluation of fundus image quality to mimic the quality diagnosis of ophthalmologists.

In the past decade, several state-of-the-art deep learning (DL) architectures, including AlexNet [4], VGGs [5], GoogLeNet [6], ResNet [7], DenseNet [8], EfficientNets [9,10], and, recently, vision transformer (ViT) [11] based models were developed for various computer vision tasks such as object localization, object detection, and classification. Even though training large DL models from scratch requires massive data, transfer learning (TL) could facilitate adapting these already trained models for new classification tasks, thus eliminating the need for huge data for retraining. Furthermore, both TL and DL have been playing a major role in healthcare by building automated diagnostic systems for several diseases using medical images from radiographs, computed tomography, digital fundus images, positron emission tomography, and magnetic resonance imaging, etc. These systems are primarily used for diagnostic and prognostic tasks and also assist medical practitioners in several scenarios such as faster data acquisition and quality control [12,13,14]. *EfficientNetV2* is one of the recently developed DL architectures based on progressive learning with a combination of training-aware neural architecture search and compound scaling to improve both the training speed and parameter efficiency [9], and it outperformed several previous state-of-the-art models including ViTs in image classification tasks on the ImageNet challenge. Therefore, the following are the contributions of this work:

- A fully automated method for the overall QE of digital fundus images is proposed using an ensemble of pretrained *EfficientNetV2-* small (*S*), medium (*M*), and large (*L*) models since model ensembling was effective in some previous studies [15,16].
- The proposed ensemble model is cross-validated and tested on a large publicly available dataset called the Deep Diabetic Retinopathy Image Dataset (DeepDRiD), as the QE of fundus images from this dataset seems challenging [3].
- The ability of the proposed ensemble model for overall QE is further stratified concerning DR disease severity.

### Related Work

Several works related to machine learning and deep learning techniques are available in the literature for the QE of digital fundus images. These works are primarily divided into two-class classification and three-class classification problems which are given in Table 1. In two-class classification, the images are divided into either good or bad quality. Whereas in the three-class problem, the images are divided into good, moderate, and bad quality. In [17], a partial least square (PLS) classifier was developed based on handcrafted features, and the method achieved an area under the receiver operator characteristic curve (AUC) of 95.8% on their private dataset. Further, a support vector machine (SVM) classifier from a mixture of private and public datasets containing fundus images of varying resolutions, Ref. [18] demonstrated an accuracy of 91.4%, Ref. [19] obtained an AUC of 94.5%, and Ref. [20] achieved a sensitivity of 95.3% in fundus image QE. In other studies, based on EyePACS Kaggle datasets [21,22], pre-trained deep learning models were fine-tuned for feature extraction. These extracted features were further fed to the SVM classifier to detect bad quality fundus images. The highest classification accuracy in these studies is 95.4%. Furthermore, several ML classifiers were developed using the openly available DRIMDB dataset, including gcforest and random forest regressor [23,24,25], and achieved accuracies above 88%.

Some recent studies on the three-class classification of fundus image quality using lightweight CNN [26] and an ensemble of CNNs [27] based on Kaggle datasets obtained accuracies above 85%. In the most recent study using pretrained ResNet50 [28], the fine-tuned model on a Kaggle dataset demonstrated an accuracy of 98.6%. Overall, using these private and public datasets mentioned thus far, the classification task is generally easier since the images are quite differentiable to the naked eye. However, in a recent digital fundus image QE grand challenge [3], the good and bad quality images in the DeepDRiD dataset are complicated to differentiate, and hence the highest accuracy obtained in the QE grand challenge was 69.81%. Therefore, the present study explored the effectiveness of *EfficientNetV2* models and their ensembling [9] to improve the overall performance of QE on the DeepDRiD.

**Table 1:** Previous works on assessing the fundus image quality using different machine learning and deep learning methods on various private and public datasets.

Study	Method	Dataset	Image Resolution	Performance (%)
Yu H et al. [17]	PLS classifier	Private—1884	4752 × 3168	AUC: 95.8
Yu F et al. [21]	SM + AlexNet + SVM	Kaggle—5200 (subset)	Original: 2592 × 1944 Resized: 256 × 256	Accuracy: 95.4 AUC: 98.2
Yao Z et al. [18]	SVM	Private—3224	-	Accuracy: 91.4 AUC: 96.2
Welikala RA et al. [20]	SVM	UK Biobank—800 (subset)	2048 × 1536	Sensitivity: 95.3 Specificity: 91.1
Wang S et al. [19]	SVM	Private and Public—536	Private: 2560 × 1960 Public: 570 × 760 and 565 × 584	AUC: 94.5 Sensitivity: 87.4 Specificity: 91.7
Shao F et al. [22]	DT, SVM and DL	EyePACS at Kaggle—4372	Multiple resolutions	Accuracy: 93.6 Sensitivity: 94.7 Specificity: 92.3
Sevik U et al. [23]	Several ML classifiers	DRIMDB—216	570 × 760	Accuracy: 98.1
Raj A et al. [27]	Ensemble of CNNs	FIQuA (EyePACS at Kaggle)—1500	Multiple resolutions	Accuracy: 95.7 (3-class classification)
Perez AD et al. [26]	Light-weight CNN	Kaggle—4768 (2-class) Kaggle—28,792 (3-class)	896 × 896	Accuracy: 91.1 (2-class) Accuracy: 85.6 (3-class)
Liu H et al. [25]	gcforest	DRIMDB—216 (3-class) ACRIMA—705 (2-class)	Multiple resolutions	Accuracy: 88.6 (DRIMDB dataset) Accuracy: 85.1 (ACRIMA dataset)
Karlsson RA et al. [24]	Random forest regressor	Private—787 oximetry and 253 RGB DRIMDB—216 (194 were used)	1600 × 1200 (oximetry) 3192 × 2656 (RGB) 760 × 570 (DRIMDB)	Accuracy: 98.1 (DRIMDB) ICC: 0.85 (oximetry) ICC: 0.91 (RGB)
Shi C et al. [28]	Pretrained ResNet50	Kaggle—2434 (2-class)	Multiple resolutions	Accuracy: 98.6 Sensitivity: 98.0 Specificity: 99.1
Liu R [3]	ISBI 2020 grand challenge	DeepDRiD—2000 (2-class)	Multiple resolutions	Accuracy: 69.81

DeepDRiD: Diabetic retinopathy—grading and image quality estimation challenge dataset. Particularly, the previous works on the DeepDRiD dataset are highlighted in bold. CNN: convolution neural network. ML: machine learning, DL: deep learning, PLS: partial least squares, SVM: support vector machine.

## 2. Methods

### 2.1. Dataset

In this study, an openly available dataset DeepDRiD from diabetic retinopathy—grading and image quality estimation challenge of ISBI 2020 was used [3]. The dataset consists of 2000 regular fundus images from 500 subjects (patients), where four images (two acquisitions per eye) for each patient were acquired. All the images are centered at the macula and optic disc. Table 2 presents the basic details of subsets formed from DeepDRiD for performance evaluation. The dataset is divided into Set-A, Set-B, and Set-C for the individual model’s training, validation, and testing.

**Figure 1:**
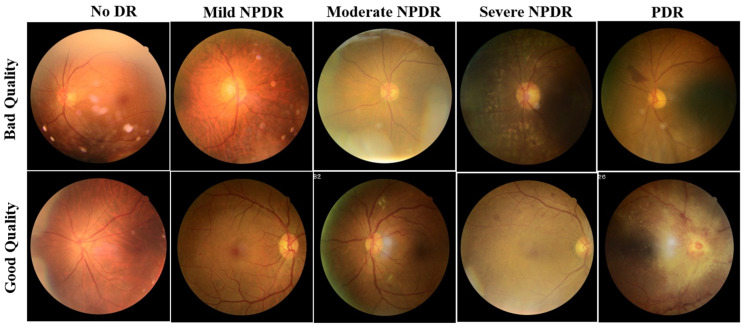
Sample fundus images of DeepDRiD dataset for good and bad quality, shown for all grades of DR. DR: diabetic retinopathy, NPDR: non-proliferative diabetic retinopathy, PDR: proliferative diabetic retinopathy.

For a fair comparison of the proposed ensemble model performance with the literature, the training, validation, and test sets in the DeepDRiD challenge remain unaltered. The images in the dataset were labelled as good and bad quality by two authorized ophthalmologists, and the labels were confirmed or revised by a third senior ophthalmologist. The example fundus images with both good and bad quality are shown in Figure 1. 

The dataset containing good and bad quality images further stratified concerning DR severity is given in Table 3 for all training, validation, and test sets. Here, by considering all 2000 images, 45.65 percent of fundus images are with no DR, 48.75 percent are with NPDR, and the rest 5.6 percent of images are with PDR.

### 2.2. EfficientNetV2

**Figure 2:**
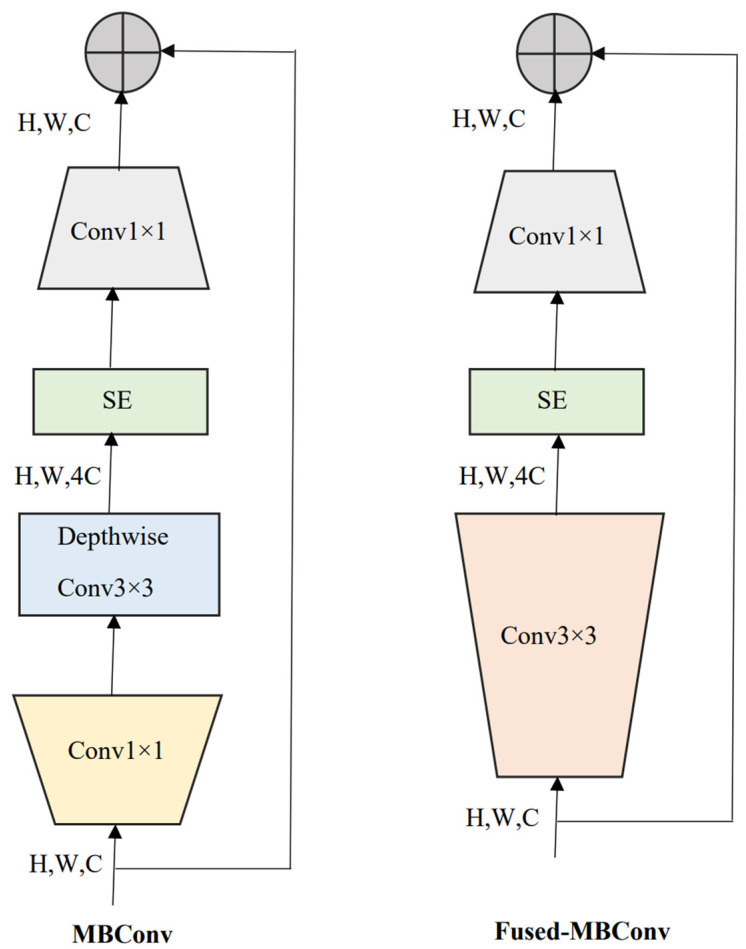
MBConv and Fused-MBConv layer architectures that were used in the family of *EfficientNetV2* models. SE: squeeze and excitation block. H, W, C: image height, width, and the number of channels.

*EfficientNetV2* [9], an improved version of *EfficientNetV1* [10], is a new family of convolutional neural networks with a special focus on two aspects: improving training speed and enhancing parameter efficiency. Towards this goal, a combination of training-aware neural architecture search and compound scaling was used. The faster training was achieved by using both MBConv and Fused-MBConv blocks. MBConv layers are basic structures of MobileNetV2 [29] built from inverted residual blocks. In the Fused-MBConv layer, two blocks (depth-wise 3 × 3 convolution and expansion 1 × 1 convolution block) in MBConv were replaced by a single (regular 3 × 3 convolution) block, as shown in Figure 2. Further, a squeeze and excitation (SE) block in MBConv and Fused-MBConv was used to adaptively weigh different channels. Finally, a 1 × 1 squeeze layer was placed to reduce the number of channels equal to the channels present in the input of MBConv/Fused-MBConv.

In the present work, we employed *EfficientNetV2-S*, *-M*, and *-L* models that use Fused-MBConv blocks in the early layers. The *EfficientNetV2-S* model architecture starts with a standard 3 × 3 convolution layer followed by three Fused-MBConv and three MBConv layers. The final layers contain a 1 × 1 convolution and maxpooling followed by a fully connected layer. Further, the *EfficientNetV2-S* model was scaled up using the compound scaling procedure to get *EfficientnetV2-M/L*. For complete details on compound scaling, refer to [9].

Furthermore, the training speed was further enhanced by progressively increasing the image size during training. However, this progressive training often results in a drop in accuracy and is prone to overfitting, which can be tackled by adaptive regularization such as dropout and data augmentation. That means weak augmentation was used for small image sizes and stronger augmentation for larger images.

### 2.3. Model Training and Validation

Initially, all the fundus images of DeepDRiD are resized to a spatial resolution of 224 × 224. Further, the model training and validation were conducted under Google Colab Pro cloud computing graphical processing unit environment with the high-level Keras API present at the backend of TensorFlow 2.0. The final classification layer of the pre-trained *EfficientNetV2-S*, *-M*, and *-L* models is removed, and an output neuron is added for the final classification of good vs. bad image quality. For this study, the hyperparameters of the models were selected empirically. The *Adadelta* optimizer with a learning rate of 0.1 was used for training, and the number of epochs was set to 10. As described in Equation (1), binary cross-entropy (*CE*) was used as the loss function since it is a 2-class classification.

(1) $$CE_{loss}=-\frac{1}{N}\overset{N}{\underset{i=0}{\sum }}ylog(\hat{y})+\left(1-y\right)log\left(1-\hat{y}\right)$$

In (1), *N* is the number of fundus images; *y* is the true label and $\hat{y}$ is the predicted label by the individual models. Only the last 20 percent of the total parameters were allowed to be fine-tuned for all individual models during training and the first 80 percent of parameters were unaltered. The validation set (Set-B) was used to make sure that the individual models were not overfitting.

### 2.4. Ensemble Model

For the ensemble model, no separate training was involved as we implemented the ensembling using the predicted probabilities of the individual models. The predicted probability of the ensemble model $p_{en}$ is calculated as the mean of the individual *EfficientNetV2-S*, *-M*, and *-L* model’s predicted probabilities $p_{s}$, $p_{m}$, and $p_{l}$, respectively. Mathematically, it is described in Equation (2).

(2) $$p_{en}=\frac{p_{s}+p_{m}+p_{l}}{3}$$

### 2.5. Evaluation Metrics

To evaluate the performances of the individual and the ensemble model, accuracy, F1-score, and balanced accuracy (*BA*) are used, which are described in Equations (3)–(5). Here, F1-scores and *BA* values which are computed from recall, specificity, and precision scores are mathematically described in Equations (6)–(8). In addition, the confusion matrix (*CM*), and the area under the receiver operating characteristic curve (AUC) are also used as model performance indicators. For example, in *CM*, given in Equation (9), *TP* is a true positive (poor image quality; label 1), *TN* is a true negative (good image quality; label 0), *FP* is a false positive, and *FN* is a false negative.

(3) $$accuracy=\frac{TP+TN}{TP+TN+FP+FN}$$

(4) $$F1-score=\frac{2*precision*recall}{precison+recall}$$

(5) $$BA=\frac{sensitivity+specificity}{2}$$

(6) $$sensitivity\;\left(recall\right)=\frac{TP}{TP+FN}$$

(7) $$specificity=\frac{TN}{TN+FP}$$

(8) $$precision=\frac{TP}{TP+FP}$$

(9) $$CM=\left[\begin{array}{cc} TP & FN\\ FP & TN \end{array}\right]$$

## 3. Results and Discussion

Table 4 presents the complete performance details of individual and ensemble models. As anticipated, the ensemble model performs better than the individual *EfficientNetV2-S*, *-M*, and *-L* models with an accuracy of 75.0 percent and an AUC of 74.9 percent on the test dataset. Among the individual models, *EfficientNetV2-L* showed better performance. Further, the performance scores of the individual models and their ensembling for the QE concerning DR grades are also presented in Table 5 in detail. The accuracy and AUC for QE of fundus images with PDR are 90.0 and 83.3 percent, respectively. In general, the performance metrics for QE are better for fundus images with PDR than those with NPDR (mild, moderate, and severe) and no DR.

**Figure 3:**
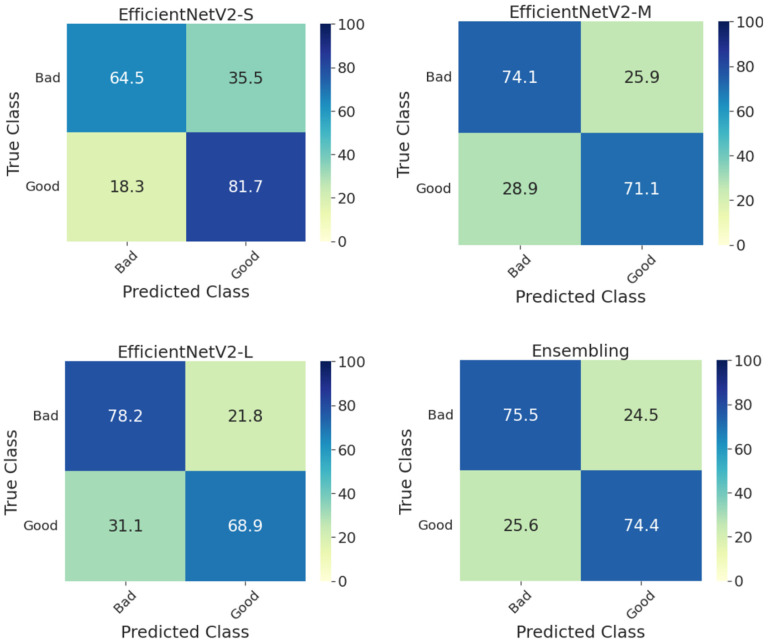
Confusion matrices of the whole test set for predicting the quality of digital fundus images using the individual and the ensemble of *EfficientNetV2-S*, *-M*, and *-L* models. *S*: small, *M*: medium, *L*: large.

**Figure 4:**
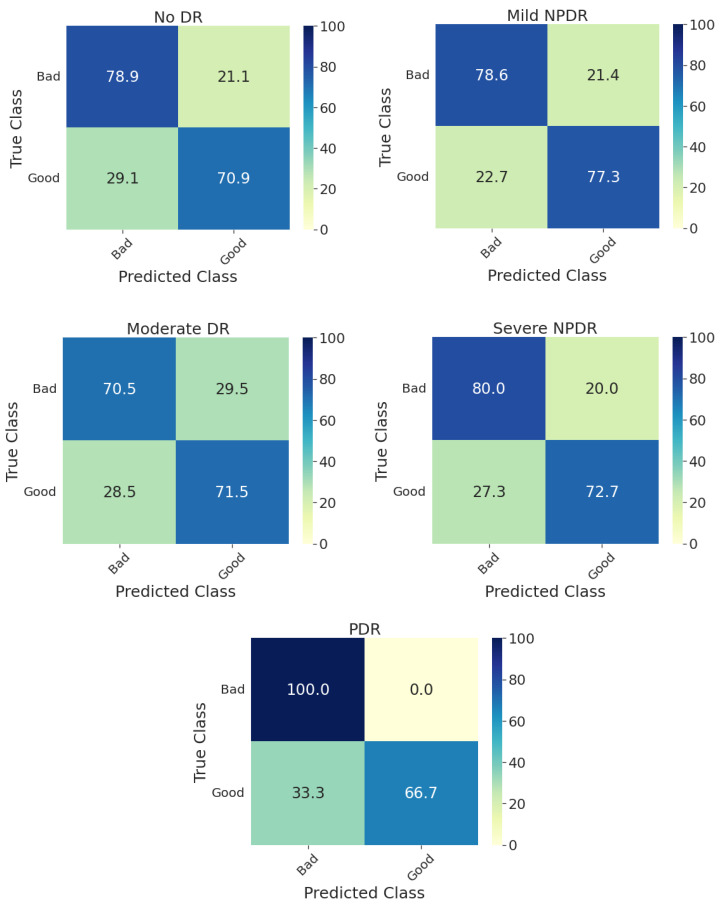
Confusion matrices of the test set for predicting the quality of digital fundus images stratified with respect to DR severity using the ensemble model. DR: diabetic retinopathy, NPDR: non-proliferative diabetic retinopathy, PDR: proliferative diabetic retinopathy.

Furthermore, Figure 3 shows the confusion matrices on the whole test set for individual models and their ensembling. Compared with the methods presented in the DeepDRiD grand challenge 2 for QE [3], our proposed ensemble model has achieved an overall accuracy of 75.0 percent, which is more than five percentage points indicating the improved robustness using our method as well as the power of ensembling. In addition, the confusion matrices for the ensemble model on the test set stratified for DR severity are given in Figure 4. In general, the method worked well for PDR images compared to the rest. For PDR images, the ensemble model has achieved 100 percent sensitivity as can be seen from the respective *CM* in Figure 4. In addition, the sensitivity is approximately 80 percent for fundus images with no DR and mild and severe NPDR. Another important aspect to observe is that the accuracy metric is typically less reliable since the labels are imbalanced in Set-C, especially for all NPDR and PDR cases as can be seen in Table 3. However, to correct for this we have employed specific performance metrics like F1-score and BA and from Table 5 we can see that these scores are very close to the accuracy values indicating that the proposed model indeed is effective in QE of fundus images.

Compared with previous studies outside the DeepDRiD on the QE of fundus images, the QE of DeepDRiD images is quite challenging since there are minimal visual differences between good and bad quality images, as can be seen in Figure 1. Further, this study demonstrates the QE with respect to DR severity that was not implemented so far to our knowledge. Moreover, in Table 1, the very high-performance metric values of various models could be because the fundus images from DRIMDB, ACRIMA, and other Kaggle datasets are quite easily differentiable to the naked eye. However, this was not the case for DeepDRiD. In addition, we suggest that the predicted probability of the proposed individual or the ensemble model can be used as the indirect measure of the estimated quality of the fundus image.

### Limitations

The size of Set-C is relatively small when the results concerning DR severity are stratified. The proposed ensembling method should be tested on other larger datasets that are similar to DeepDRiD to corroborate the ability of the proposed method for QE and there exists scope for improvement. Although the individual *EfficientNetV2* model hyperparameters were empirically chosen, a more thorough search of hyperparameters, including the optimizer’s choice, may be performed via grid or random search. Nevertheless, based on a few experiments conducted, *Adadelta* worked better in terms of overall accuracy than other well-known optimizers including *RMSprop* and *Adam*. Further, it would be interesting to add explainability to the proposed model to better understand its decisions and to identify the degraded regions in the bad quality fundus images. We would like to explore this direction in a future study.

## 4. Conclusions

In this study, we have proposed a framework for QE of digital fundus images using *EfficientNetV2-S*, *-M*, and *-L* models. The ensemble model presented in this study has achieved an accuracy of 75.0 percent and an AUC of 74.9 percent on the whole test set for QE. The performance is better than the existing works for QE of fundus images from the DeepDRiD. Further, the performance metrics of QE are generally superior for images with PDR than all NPDR and no DR. Hence, the proposed ensemble model could assist ophthalmologists by automating the QE of the fundus image before proceeding with DR severity grading. The code for this study could be provided upon reasonable request.

## Figures and Tables

**Table 2:** Details of training, validation, and test set formed from DeepDRiD regular fundus images. BMI: body mass index.

	No. of Images	No. of Subjects	Female (%)	Age (Years)	BMI (kg/m^2^)
Set-A (training)	1200	300	49.00	70.63 ± 7.70	25.17 ± 3.13
Set-B (validation)	400	100	56.00	65.13 ± 1.89	24.88 ± 3.21
Set-C (testing)	400	100	54.00	61.36 ± 7.23	25.01 ± 2.6

**Table 3:** The number of good and bad quality images in the training, validation, and test set stratified with respect to DR severity. DR: diabetic retinopathy, NPDR: non-proliferative diabetic retinopathy, PDR: proliferative retinopathy.

	No DR	Mild NPDR	Moderate NPDR	Severe NPDR	PDR
Set-A (Training)	Good: 234 Bad: 306	Good: 74 Bad: 66	Good: 126 Bad: 108	Good: 108 Bad: 106	Good: 34 Bad: 38
Set-B (Validation)	Good: 62 Bad: 112	Good: 32 Bad: 14	Good: 48 Bad: 44	Good: 30 Bad: 38	Good: 10 Bad: 10
Set-C (Testing)	Good: 86 Bad: 113	Good: 22 Bad: 14	Good: 44 Bad: 28	Good: 22 Bad: 50	Good: 6 Bad: 14

**Table 4:** Performance metrics for QE of all test set images for individual *EfficientNetV2* models and their ensembling (all the values are in percentages). BA: balanced accuracy, AUC: area under the curve, QE: quality estimation.

	EfficientNetV2-S	EfficientNetV2-M	EfficientNetV2-L	Ensemble Model
Accuracy	72.3	72.8	74.0	75.0
AUC	73.1	72.6	73.5	74.9
F1-Score	72.2	72.8	73.9	75.0
BA	73.1	72.6	73.5	74.9

**Table 5:** Performance metrics for QE of test images stratified concerning DR severity for individual *EfficientNetV2* models as well as their ensembling. BA: balanced accuracy, AUC: area under the curve, QE: quality estimation, DR: diabetic retinopathy, NPDR: non-proliferative diabetic retinopathy, PDR: proliferative diabetic retinopathy.

		EfficientNetV2-S	EfficientNetV2-M	EfficientNetV2-L	Ensemble Model
No DR	Accuracy	71.5	73.0	72.5	75.5
	AUC	72.3	72.7	71.5	74.9
	F1-Score	71.6	73.0	72.3	75.5
	BA	72.3	72.7	71.5	74.9
Mild NPDR	Accuracy	72.2	77.8	75.0	77.8
	AUC	69.5	76.2	73.1	77.9
	F1-Score	71.8	77.8	74.8	78.0
	BA	69.5	76.2	73.1	77.9
Moderate NPDR	Accuracy	70.2	70.5	70.6	71.0
	AUC	70.5	70.8	70.8	71.8
	F1-Score	70.1	70.1	71.1	71.2
	BA	70.5	70.9	70.8	71.5
Severe NPDR	Accuracy	76.4	72.2	77.8	77.8
	AUC	79.2	68.5	73.8	76.4
	F1-Score	77.3	72.5	77.8	78.2
	BA	79.2	68.5	73.8	76.4
PDR	Accuracy	70.0	90.0	85.0	90.0
	AUC	69.0	83.3	75.0	83.5
	F1-Score	71.0	89.3	83.2	89.3
	BA	69.0	83.3	75.0	83.5

## Data Availability

The dataset used in this study is publicly available.
